# The Chemical Action of Strychnine, Quinine, Aconitine, and Atropine

**Published:** 1895-09

**Authors:** A. D. Barr

**Affiliations:** Calamine, Ark.


### *CORRESPONDENCE.*

---

### THE CHEMICAL ACTION OF STRYCHNINE, QUININE, ACONITINE, AND ATROPINE.

CALAMINE, ARK., August 1, 1895.

*Atlanta Medical and Surgical Journal:*

In order to intelligently understand the physiological action of drugs, it is necessary to know what chemical changes, if any, they are subjected to in the system.

It is evident that the mere presence of a foreign substance in the system is not sufficient to account for the energetic action of the more potent remedies.

That the action of drugs is not due to the ordinary process of oxidation (as of the carbohydrates, etc.) is proven by these facts: First. That the heat is generated too slowly in the ordinary process of oxidation to account for the action of the more powerful agents. Second. If this were so the effect of all drugs oxidized would vary in effect in *degree* only. Third. As the oxidation process takes place almost, if not entirely, in the epithelial structure of the various glandular organs of the body, including the follicular structure of the alimentary canal, the action on different organs cannot thereby be accounted for.

It is to chemistry that we must look for the explanation of the physiological action of all therapeutic agents. It is the power that organic bodies possess of entering into combination as a whole with other chemical compounds, that gives the explanation of the physiological action of drugs, rather than any decomposition and formation of new chemical compounds.

That the physiological action of strychnine, quinine, aconitine, and atropine is due to chemical action and heat generated thereby is proven by the following experiment: Rub a brain to a paste in a mortar, then add a solution of one of the above named drugs, and a rise of temperature will occur, varying from one to three or more degrees F., thus demonstrating chemical combination. If the gray matter alone is used, no perceptible rise will occur. The white matter of the brain causes by far the greatest rise, and certain portions greater elevations than others. The same effect is noticed with the spinal cord. The medulla produces the most rapid and the greatest rise of any portion of the brain. Strychnine causes an almost instantaneous rise, which continues for some little time; while with quinine there is no rise for some time, and when the rise does occur, it is decidedly more slow and continues for some hours. Aconitine causes the greatest rise of temperature in the same length of time of any of the drugs I have experimented with; its action may be compared to an explosion, the rise of temperature is so rapid. Atropine causes a decided and rapid rise of temperature, about the same as strychnine. In these experiments it is far best to use a calorimeter, but if care be taken to bring the brain and the solution of the drug to the *exact* temperature of the air, with such drugs as these, a decided rise of temperature will occur.

I have succeeded in obtaining a rise of three degrees in an external temperature several degrees lower than that of the substance experimented upon. Just what the chemical combination is that takes place I am not at present prepared to say; but as all these substances are bases, and as their salts are converted in the system into the original base, it is almost conclusive that the heat is generated by the combination of the basic alkaloid with a radicle in the nervous system, and the radicle is probably derived from lecithin, as it is a glycero-phosphoric acid, and strychnine is eliminated from the system as acid strychnine.

The force generated in the chemical combination as evinced by the rise of temperature is sufficient to explain the action of the drugs that have been considered, especially when it is remembered that the force is generated in as delicate a structure as the nervous system.

A. D. BARR, M.D.

